# Do mobile phone-based reminders and conditional financial transfers improve the timeliness of childhood vaccinations in Tanzania? Study protocol for a quasi-randomized controlled trial

**DOI:** 10.1186/s13063-019-3430-4

**Published:** 2019-07-04

**Authors:** Jan Ostermann, Lavanya Vasudevan, Joy Noel Baumgartner, Esther Ngadaya, Sayoki Godfrey Mfinanga

**Affiliations:** 1 Department of Health Services Policy & Management, Arnold School of Public Health, University of South Carolina, Columbia, SC 29208 USA; 2 Duke Global Health Institute, Durham, NC 27708 USA; 3 Department of Family Medicine and Community Health, School of Medicine, Duke University, Durham, NC 27705 USA; 4 Muhimbili Research Centre, National Institute for Medical Research, Dar-es-Salaam, Tanzania

**Keywords:** Vaccinations, Vaccination timeliness, Child health, Mobile phones, Targeted client communication, Transmit targeted alerts and reminders to clients, Short message service (SMS), Conditional financial transfers, Client financial transactions, Transmit or manage incentives to clients for health services, Tanzania, Sub-Saharan Africa

## Abstract

**Background:**

Vaccination is a cost-effective strategy for reducing morbidity and mortality among children under 5 years old. To be fully protected from diseases such as tuberculosis, diphtheria, pertussis, and polio, children must receive all recommended vaccinations in a timely manner. In many countries, including Tanzania, high overall vaccination rates mask substantial regional variation in vaccination coverage and low rates of vaccination timeliness. This study evaluates the efficacy of mobile phone-based (mHealth) reminders and incentives for improving vaccination timeliness in the first year of life.

**Methods:**

The study, conducted in Mtwara Region in Tanzania, includes 400 late-stage pregnant women enrolled from rural and urban health facilities and surrounding communities. The primary outcome is timeliness of vaccinations among their children at 6, 10, and 14 weeks after birth. Timeliness is defined as vaccination receipt within 28 days after the vaccination due date. The quasi-randomized controlled trial includes three arms: (1) standard of care (no reminders or incentives), (2) mobile phone-based reminders, and (3) mobile phone-based reminders and incentives in the form of conditional financial transfers. Assignment into study arms is based on scheduled vaccination dates. Reminder messages are sent to arms 2 and 3 participants via mobile phones 1 week and 1 day prior to each scheduled vaccination. For arm 3 participants, reminder messages offer an incentive that is provided in the form of a mobile phone airtime recharge voucher code for each timely vaccination. Vaccination dates are recorded via participant contact with an mHealth system, phone calls with mothers, and a review of government-issued vaccination cards during an end-line survey. Random effects logistic regression models will be used to estimate the effects of reminders and incentives on the timeliness of vaccinations.

**Discussion:**

The results will inform implementation science research on the effectiveness of reminders and incentives as a means of improving vaccination timeliness.

**Trial registration:**

ClinicalTrials.gov, NCT03252288. Registered on 17 August 2017 (retrospectively registered).

**Electronic supplementary material:**

The online version of this article (10.1186/s13063-019-3430-4) contains supplementary material, which is available to authorized users.

## Background

Globally, childhood vaccinations are estimated to prevent 2.5 million deaths annually [1]. Yet, 19.7 million children under the age of 1 year have missed receiving basic vaccinations [1]. The majority of un- or under-vaccinated children reside in low- and middle-income countries (LMICs), with those from rural, remote, and poorer households being disproportionately less likely to receive all recommended vaccines in their first year of life [1–3]. In Tanzania, for example, 1 in 4 children has not received all recommended vaccines in the first year of life, compared to 1 in 10 globally [4, 5]. Significant regional variation in vaccination coverage has been reported in Tanzania, with several regions failing to meet the 80% coverage target set forth in the Global Vaccine Action Plan [5–7]. Furthermore, numerous reports indicate low timeliness of vaccinations in LMICs, including in Tanzania [8–10]. In a study in rural Tanzania, vaccination delays were reported in 69% of children for the third dose of the diphtheria-pertussis-tetanus (DPT) vaccine and in 46% of children for the measles-containing vaccine (MCV) [10]. In the quest for universal immunization, innovative strategies are needed to improve vaccination coverage for the “last mile” and to reduce vaccination delays that leave children susceptible to unnecessary morbidity and mortality from vaccine-preventable infections [2].

Barriers to timely immunization are multifaceted and include immunization system constraints (e.g., vaccine stock-outs, limited availability of health care providers), poor access to health facilities (e.g., transport cost, opportunity cost), lack of information (e.g., about the vaccination schedule or community mobilizations), and negative parental attitudes and inadequate knowledge (e.g., lack of trust in vaccinators, poor knowledge of vaccination benefits, fear of vaccination side effects) [3, 11, 12]. In recent years, many groups have leveraged the global pervasiveness of mobile phones for alleviating some of these barriers in order to improve vaccination coverage and timeliness in LMICs [13, 14]. Implementations of mobile phone-based (“mHealth”) strategies in the context of vaccinations include birth notification and newborn surveillance for early identification of children eligible for vaccination services [15–17]; development of digital vaccination registries to longitudinally track the vaccination status of children and rapidly identify those who are unvaccinated or under-vaccinated [17–23]; use of text messages (SMS) or simple voice communication to deliver health promotion messages and reminders about upcoming vaccination appointments [17, 24–26]; delivery of incentives for timely vaccinations [27–29]; digital aids to assist vaccinators in counseling parents [23], for work planning and scheduling [17, 18, 23], or for vaccine stock management [30]; and curation of performance indicators at the regional and national levels [31]. Despite these numerous instantiations, the evidence base on the efficacy of mHealth strategies for improving childhood vaccination coverage and timeliness is limited, with the need for rigorous evaluations identified in several systematic reviews [14, 32, 33].

With support from the National Immunization and Vaccines Development (IVD) program of the Tanzanian National Ministry of Health, Community Development, Gender, Elderly and Children, we are evaluating the efficacy of mobile phone-based vaccination reminders and incentives in the form of conditional financial transfers (CFTs) for offsetting demand-side barriers to timely vaccination. Per the recently released World Health Organization (WHO) classification of digital health interventions, the two key interventions in this study correspond to 1.1 Targeted client communication (specifically, 1.1.3 Transmit targeted alerts and reminders to clients) and 1.7 Client financial transactions (specifically, 1.7.3 Transmit or manage incentives to clients for health services) [34]. Our hypothesis is that reminders and incentives will increase timeliness of vaccinations relative to the status quo. The design and protocol of the evaluation are described in the following sections.

## Methods/design

### Study aim

The aim of the study is to assess the efficacy of (1) mobile phone-based reminders, and (2) mobile phone-based reminders combined with incentives in the form of CFTs, for improving vaccination timeliness among children at 6, 10, and 14 weeks after birth, compared to the standard of care.

### Outcome measures

The recommended schedule for vaccinations by age 1 year is presented in Table 1. The primary study outcome is whether or not a child received an on-time vaccination; the outcome is measured for vaccinations scheduled at 6, 10, and 14 weeks after birth. Secondary outcomes include vaccination coverage, in aggregate and for each of the vaccinations due at 6, 10, and 14 weeks after birth. Timeliness is defined as vaccination within 0-28 days after the scheduled vaccination due date. Coverage is defined as the percentage of children vaccinated.

**Table 1:** Recommended vaccinations by age 1 year in Tanzania

Target age	Recommended vaccines
At or soon after birth	OPV(0), BCG
6 weeks	OPV(1), Penta(1), PCV(1), Rota(1)
10 weeks	OPV(2), Penta(2), PCV(2), Rota(2)
14 weeks	OPV(3), Penta(3), PCV(3)
9 months	MR

Abbreviations: *OPV* oral polio vaccine, *BCG* Bacillus Calmette-Guerin vaccine; *Penta* pentavalent vaccine (diphtheria, pertussis, tetanus, *Haemophilus influenzae* type b, hepatitis B), *PCV* pneumococcal vaccine, *Rota* Rotarix (vaccine against rotavirus), *MR* Measles-rubella vaccine

### Study design

**Fig. 1:**
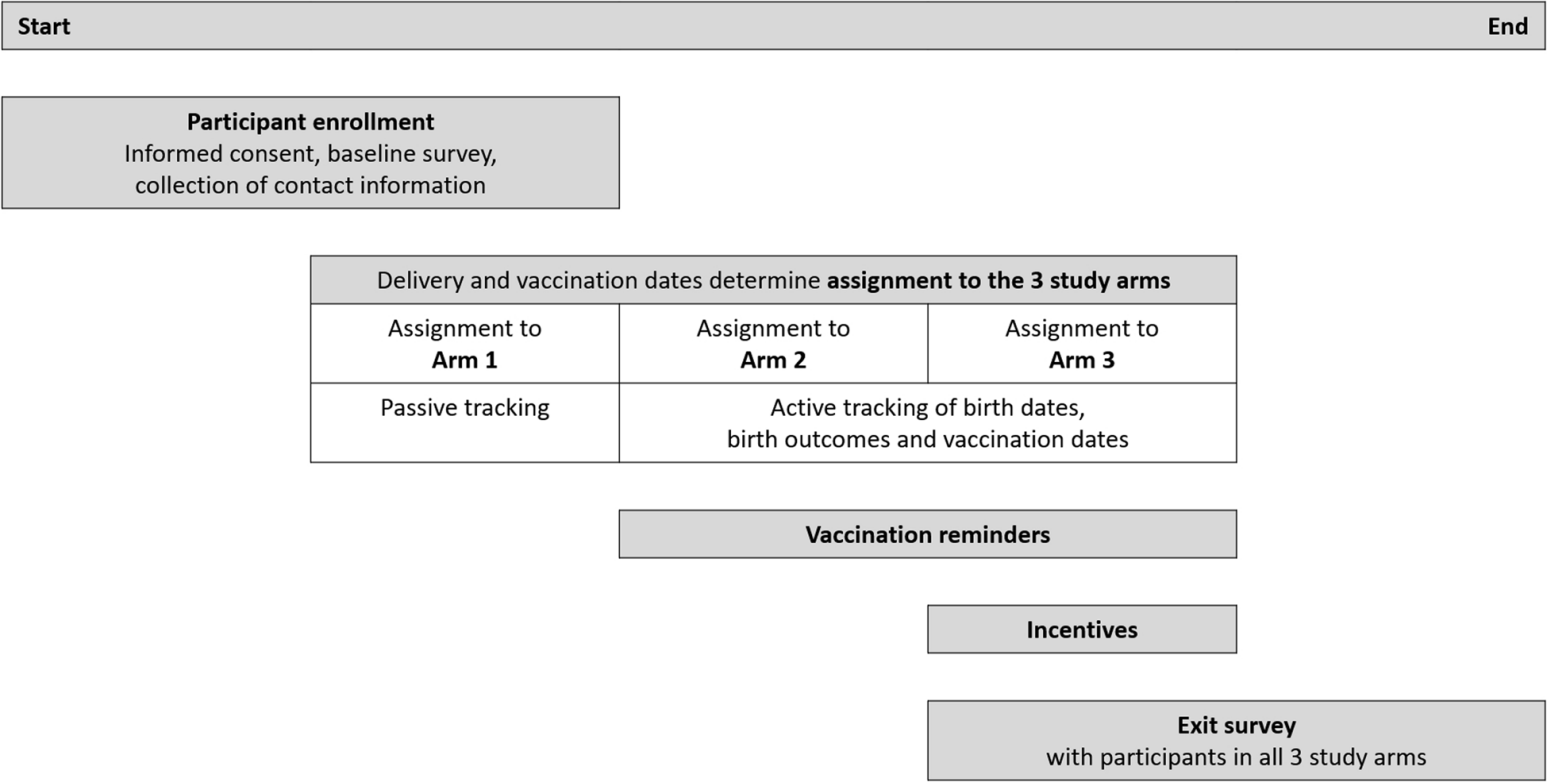
Sequence of events for the quasi-randomized controlled trial

The study design represents a quasi-randomized controlled trial that includes three phases, each corresponding to one study arm: (1) standard of care (no reminders or incentives), (2) mobile phone-based reminders, and (3) mobile phone-based reminders combined with incentives in the form of CFTs. Arm 1 serves as the control arm; arms 2 and 3 are intervention arms. The trial is enfolded by baseline and end-line assessments (Fig. 1).

### Assignment to study arms and transitions between arms

Assignment to study arms is implemented at the level of the vaccination event, with all vaccination events scheduled during a phase assigned to the corresponding arm. T_1_, the beginning of Phase 1, denotes the time of participants’ enrollment into the control arm. At time T_2_, the beginning of Phase 2, all participants are rolled over to the arm with reminders; at time T_3_, the beginning of Phase 3, all participants are rolled over to the arm with reminders combined with incentives. While T_2_ and T_3_ denote fixed points in calendar time, variation in delivery dates across participants results in between- and within-individual variation in the assignment of vaccination events across the three arms. Table 2 shows all possible combinations of vaccination event assignments to study arms at the level of the participant. With dates of birth exogenously determining children’s vaccination schedules, shifts in T_2_ and T_3_ directly affect the number of vaccination events that may be observed in each study phase. Based on the distributions of expected delivery and vaccination due dates, T_2_ and T_3_ are chosen such that the expected number of vaccination events is approximately equal across the three arms. With T_2_ and T_3_ being random in relation to mothers’ delivery dates, and thus children’s vaccination schedules, the assignment of vaccination events across arms may be considered quasi-random. Loss to follow-up and other reasons for non-delivery of vaccination reminders result in exposure indistinguishable from the control condition.

**Table 2:**
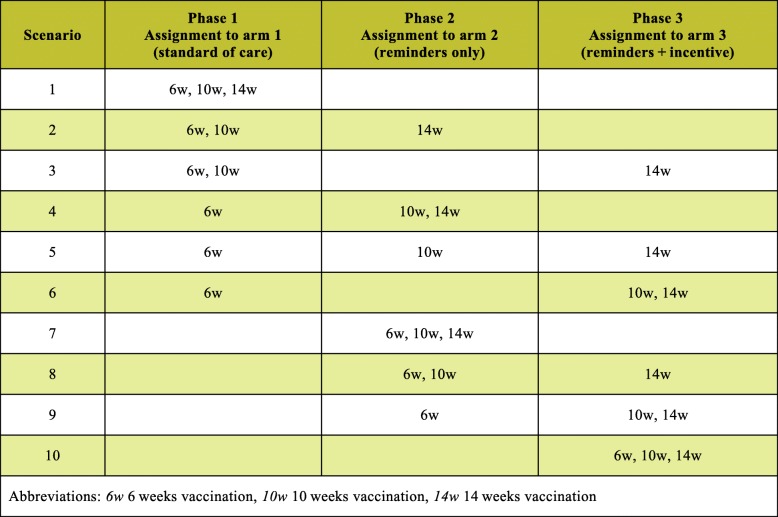
Possible combinations of vaccination assignments to study arms at the level of the participant

The assignment to study arms based on scheduled vaccination dates and the simultaneous transition of all participants between study arms address concerns that the individual randomization into, and co-existence of, incentive and non-incentive arms are not considered viable within closely knit communities. While cluster randomization was considered as an alternative approach, the heterogeneity of clusters suggests that this approach would confound the effects of settings with the effects of reminders and incentives.

### Study setting

The study is conducted in two adjacent districts, one urban district (Mtwara Municipality) and one rural district (Mtwara District Council), in Mtwara Region in Southeastern Tanzania. In 2017, Mtwara Municipality had an estimated population of 115,132; Mtwara District Council had an estimated population of 130,757 [35]. Owing to logistical considerations, the study area in the rural district is limited to a 30-km radius around the urban district.

### Study participants

Eligible study participants are women, ages 16 or older, who are in the third trimester of pregnancy and have access to a mobile phone. The target enrollment is 400 women.

### Recruitment

Participants are recruited from at least four urban and at least eight rural health facilities and the surrounding communities. Eligible health facilities regularly provide childhood vaccinations. A combination of purposive and snowball sampling strategies is used for recruitment as follows: eligible women presenting for antenatal care at participating facilities are approached by trained study personnel and offered enrollment in the study. To minimize biases from facility-based enrollment, participating women and local community leaders (*balozis*) are asked to identify other pregnant women in their community, who, if eligible, are also offered enrollment in the study.

### Enrollment and informed consent

All individuals contacted for participation in the study are informed of the study purpose and procedures, as well as the risks and benefits, during the informed consent process by study staff. Women are approached in person and are provided study information orally and in writing using an informed consent script in Kiswahili. Only consenting women are included in the study. Some women may access mobile phones via relatives, friends, or other designees: these persons are considered authorized agents for the purpose of this study. Authorized agents are not considered research subjects and, thus, are not independently consented. By designating authorized agents, participating women are considered to be providing implied consent for these agents to receive information on their behalf.

Since the study includes participants ages 16 years and older, the Institutional Review Board (IRB) granted a waiver of parental consent for pregnant women ages 16 and 17 years old. Tanzanian national policy indicates that adolescents under age 18 who are sexually active are considered “mature minors”; they have the right to access reproductive health services without parental or spousal consent, and they can make associated medical decisions on their own behalf [36]. Given that sexually active adolescents have this “adult” right to medical decision-making, it was determined that pregnant adolescents ages 16 and 17 years are also able to consent to research participation for themselves without parental or spousal consent.

### No blinding

The nature of the interventions, mobile phone-based reminders and incentives in the form of CFTs, precludes blinding of study participants.

### Intervention components and implementation

For the delivery of the study interventions, we developed an mHealth system comprising several components, as described in the following subsections.

#### Scheduling system

Upon enrollment, a participant’s name, contact information, and expected delivery date (EDD) are stored in a database, and participants are added to a process queue. For each participant, the process awaits inputs (e.g., date of birth of the child, vaccination dates) and executes tasks according to a prespecified schedule. Tasks include sending reminder SMS messages, tracking SMS delivery, and alerting study staff to call participants when follow-up action is required. When event-related inputs (e.g., dates of completed or rescheduled vaccinations) are received by the system, the database and the schedule of tasks are updated. For example, a delay in one vaccination shifts the remaining schedule by the length of the delay.

#### Unstructured Supplementary Service Data (USSD) notification system

Participants can notify the scheduling system of events (e.g., birth of child, vaccinations received) using an Unstructured Supplementary Service Data (USSD) dialog. USSD dialogs, which can be accessed free of charge by dialing a short code from any mobile phone, allow for a two-way exchange of data. In this case, the USSD dialog is implemented analogously to a short survey. Participants are guided through text-based menus which prompt them for information related to the event they are reporting. The information is passed to the scheduling system, which updates the schedule of upcoming tasks.

#### Mobile phone reminder system

Participants in arms 2 and 3 receive reminders for upcoming vaccination appointments 1 week and 1 day before the vaccination due date. No reminders are associated with the EDD or the vaccination due at birth. Reminders are issued in the form of text messages to the mobile phone numbers of study participants or their designees. Prior to study implementation, messages were tested for comprehension using a structured template, and revised based on feedback received. The timing of messages is based on findings from a cross-sectional survey of 134 Tanzanian mothers of children ages 12–23 months. In that survey, 54% of women who indicated any mobile phone use wanted to receive reminders 1 week before the due date, 40% 1 day before the due date, and 6% on the due date. Data on comprehension are collected in the end-line survey to inform future implementations.

#### Incentive disbursement system

For each timely vaccination for which a reminder with an incentive offer was sent to a mobile phone, a payment in the form of a mobile phone airtime recharge voucher code (worth either TSH 1000 or TSH 2000) is sent to a mobile phone number specified by the participant. The two-tiered incentive amount, which was chosen ad hoc by the study team as appropriate to “nudge” participants toward timely vaccinations without significantly changing their economic incentives [37], is conditional upon the timing of vaccinations (see the following sections).

#### Technical implementation

The scheduling and sending of SMS messages and incentives is implemented via a custom-built task scheduling, execution, and event tracking application called mParis (“mobile phone assisted reminder and incentive system”). mParis, which was developed for another National Institutes of Health (NIH)-funded study (R01MH106388), is built using Flowable, a flexible, open source, Java-based, workflow engine (https://www.flowable.org) [38]. The system is based at the Kilimanjaro Clinical Research Institute, in Moshi, Tanzania.

### Study activities

**Fig. 2:**
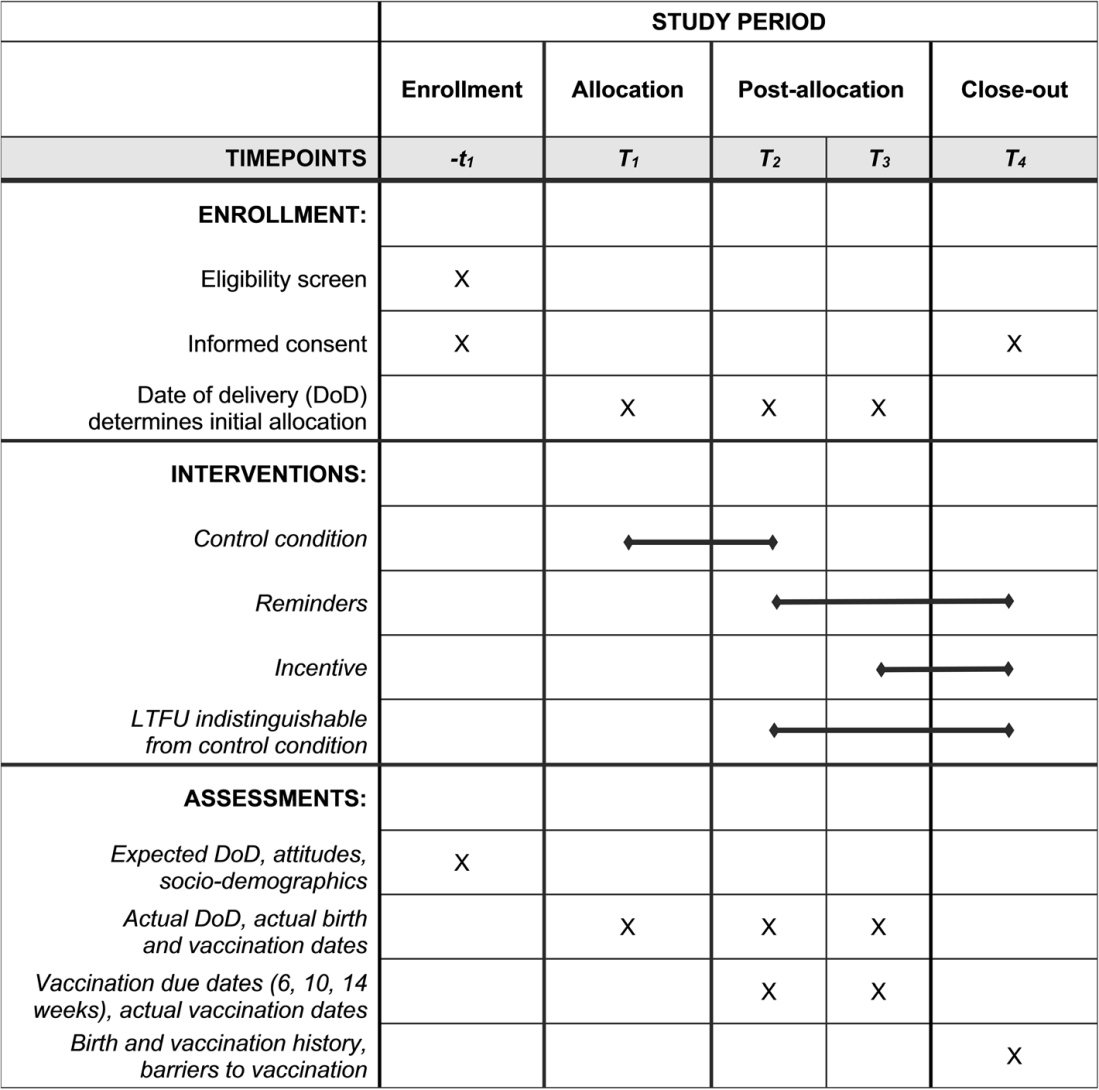
SPIRIT figure depicting schedule of enrollment, interventions, and assessments

Key events and related study activities are summarized in Figs. 1 and 2 and Table 3.

**Table 3:** Study events and activities

	Arm 1 (standard of care)	Arm 2 (reminders only)	Arm 3 (reminders + incentive)
Pregnancy	Recruitment and enrollment during the last trimester (EDD within 3 months). In-person baseline survey		
Birth		Phone-based documentation of birth date and birth outcome within 14 days of EDD	
Vaccination V_0_ *At birth (t*_*0*_ *)* *BCG* *OPV(0)*		Phone-based confirmation of V_0_ visit at *t*_0+14d_. If not vaccinated, follow up every 14 days Updates to *t*_6w_, *t*_10w_, and *t*_14w_ as needed	
			Full amount if V_0_ visit by *t*_0+14d_ Half amount if V_0_ visit between *t*_0+14d_ and *t*_0+28d_ None after *t*_0+28d_
Vaccination V_1_ 6 *weeks (t*_*6w*_*)* *OPV(1)* *Penta(1)* *PCV(1)* *Rota(1)*		Reminders at *t*_6w-7d_ and *t*_6w-1d_ Phone-based confirmation of V_1_ visit at *t*_6w+14d_. If not vaccinated, follow up every 14 days Updates to *t*_10w_ and *t*_14w_ as needed	
			Full amount if V_1_ visit by *t*_6w+14d_ Half amount if V_1_ visit between *t*_6w+14d_ and *t*_6w+28d_ None after *t*_6w+28d_
Vaccination V_2_ *10 weeks (t*_*10w*_ *)* *OPV(2)* *Penta(2)* *PCV(2)* *Rota(2)*		Reminders at *t*_10w-7d_ and *t*_10w-1d_ Phone based confirmation of V_2_ visit at t_10w+14d_. If not vaccinated, follow up every 14 days. Updates to t_14w_ as needed	
			Full amount if V_2_ visit by t_10w+14d_ Half amount if V_2_ visit between t_10w+14d_ and t_10w+28d_ None after t_10w+28d_
Vaccination V_3_ 14 *weeks (t*_*14w*_*)* *OPV(3)* *Penta(3)* *PCV(3)*		Reminders at t_14w-7d_ and t_14w-1d_ Phone based confirmation of V_3_ visit at t_14w+14d_. If not vaccinated, follow up every 14 days.	
			Full amount if V_3_ visit by t_14w+14 days_ Half amount if V_3_ visit between t_10w+14d_ and t_10w+28d_ None after t_14w+28d_
Exit survey	In-person survey to assess barriers to vaccinations, acceptability of reminders (arms 2 and 3) and incentives (arm 3), and document vaccination coverage and timeliness using government-issued vaccination cards		

Abbreviations: EDD = expected date of delivery; w = weeks; d = days; t = time; BCG, OPV, Penta, PCV, Rota: see Table 1

Women providing informed consent are enrolled in the study. At the time of enrollment, a baseline survey is conducted with all participants to assess socio-demographic characteristics, mobile phone ownership and use, reproductive history, EDD for the current pregnancy, vaccination knowledge, history of vaccinations in a prior child, if applicable, and previously experienced and/or anticipated barriers to vaccinations. Mobile phone numbers are obtained for mothers and any authorized agents. Surveys are implemented electronically on tablet devices and conducted by study staff at health facilities, participants’ homes, or other mutually agreed-upon locations.

Toward the end of Phase 1, all women are contacted by phone to ascertain birth dates and outcomes, and information on past and scheduled vaccinations. The information is used to generate child-specific vaccination schedules. In Phases 2 and 3, reminder messages are sent to registered mobile phone numbers 1 week and 1 day prior to each scheduled vaccination, and, if no vaccination confirmation is received, 2 weeks after the vaccination due date. Uniquely numbered cards, given to participants at each completed or rescheduled vaccination, allow participants to report vaccination dates via the USSD system. When no vaccination report is received via the USSD system, SMS, or phone calls from participants, study staff contact the participant by phone to verify whether and when a vaccination was received and to ascertain the due date for the next vaccination, if applicable. Recorded vaccination dates are used to iteratively update vaccination schedules and to reschedule reminders. Additional file 1: Table S1 shows the content of SMS reminders.

During Phase 3, reminder messages offer an incentive for each timely vaccination. The full incentive amount (TSH 2000; approximately $0.90) is paid when a vaccination is received within 2 weeks (14 days) of the due date; half the incentive is paid if the vaccination is completed more than 2 weeks but within 4 weeks (28 days) after the vaccination due date. Incentive amounts are paid if the mother, despite going to the health facility as reminded, is asked to return for a vaccination at a later date. Payments for timely vaccinations are made via an SMS message that includes a network-specific mobile phone airtime recharge voucher code and is sent to a phone number specified by the mother. An in-person end-line survey, conducted 12 months after enrollment, assesses barriers to vaccinations and receipt and acceptability of reminders (arms 2 and 3) and incentives (arm 3), and records vaccination dates from participants’ government-issued vaccination cards, allowing for comparisons with the data recorded in the mHealth system.

### Data management

Password-protected tablet devices are used to collect survey data from participants. Data transfer agreements (DTAs), signed by all participating institutions, guide the secure sharing of data between Tanzania and foreign collaborators. To ensure confidentiality, identifying information is collected, transferred, and stored separately from survey data. On-site study monitoring, performed by the Principal Investigators or their designees, is used to verify compliance with human subjects and other research regulations and guidelines, assess adherence to the study protocol, and confirm the quality and accuracy of information collected and entered into the study database. All collaborators have access to the final data.

### Statistical analysis

For each participant, outcomes are observed at up to three time points (corresponding to timely vaccinations at 6, 10, and 14 weeks, respectively). The probability of timely vaccinations and its association with reminders and incentives will be analyzed using a random effects logistic regression model. The outcome variable is 1 if the vaccination was received on time, and 0 if early, delayed, or not received at all. The primary covariates of interest are indicator variables for the receipt of reminders only (*reminder*) and the receipt of reminders with an incentive offer (*reminder + incentive*). For arm 1 the value of both covariates is 0 for all three time points. For outcomes in arm 2, the value for *reminder* (and for arm 3 the value for *reminder + incentive*) is 1 if the mHealth system confirmed delivery of the reminder, and 0 for all other observations. Additional covariates (e.g., mother’s education, rural vs. urban residence, recruitment from health facilities vs. the community) will control for differences in participant characteristics between study arms and their association with vaccination timeliness. Interactions of *reminder* and *reminder + incentive* with these covariates describe systematic variation in the effects of reminders and incentives on vaccination timeliness. Models will control for the number of phone-based contacts prior to or within the timeliness window for any given vaccination, and standard errors will be adjusted to account for clustering at the level of the health facility catchment area.

### Statistical power

Across 400 participants we expect up to 1200 data points on vaccination timeliness. Assuming (1) that these data points are distributed equally across the 3 study arms (standard of care; reminders only; reminders and incentive); (2) a 10% attrition rate; (3) equal cluster sizes of 20 women per health facility catchment area, with an intra-cluster correlation of 0.02; (4) 3 observations per child, with a within-child correlation of 0.2; (5) a baseline rate of vaccination timeliness of 65% in the control condition; and (6) a 10 (15) percentage point difference associated with reminders (reminders and incentives), the power (α = 0.05, two-tailed) to detect such a difference is 72% (98%).

### Dissemination

Results will be shared with stakeholders at the regional and national levels, presented at national and international conferences, and published in high-impact academic journals.

### Data sharing

Efforts will be made to develop a data repository for the storage of de-identified study data. Investigators wishing to use study data to answer new research questions may submit data analysis concept proposals for consideration by the Principal Investigators. The Principal Investigators will review the proposals and will provide those submitting scientifically rigorous and promising proposals access to the data repository to address their research questions.

## Discussion

This study examines the efficacy of mobile phone-based reminders and incentives in the form of conditional financial transfers for offsetting demand-side barriers to timely childhood vaccinations in Tanzania. Our implementation strategy has several unique components, including the use of a USSD-based birth and vaccination event notification system, an adaptive schedule for vaccination reminders which is derived from the date of the preceding event, a tiered incentive system, and the semi-automated disbursement of mobile phone credit for eligible timely vaccinations.

The study is subject to several limitations. First, feasibility considerations limit the study area to include only health facilities and their catchment areas within approximately 30 km of Mtwara Municipality. More remote populations may face additional barriers that cannot be evaluated in this study. Second, only women with access to mobile phones are included in the study. To minimize selection bias, women could designate anyone with a mobile phone to be their study contact. However, for women relying on such authorized agents, receipt of messages and payments for timely vaccinations could remain a challenge. Third, reminder schedules and payments for timely vaccinations are based on client-reported birth and vaccination dates. The dependence on client reports may introduce delays, affect schedule accuracy, and motivate inaccurate reporting of vaccination dates. Comprehensive quality control, including automated alerts to study staff for participant follow-up, algorithms that identify inconsistencies in near real time, and an in-person end-line survey with a review of government-issued vaccination cards, will be used to assess and/or mitigate these concerns. Fourth, it is plausible that follow-up phone calls by study staff to ascertain birth outcomes, the timing of past vaccinations, and/or the timing of the next scheduled vaccination, may themselves function as an intervention. Fifth, temporal changes that coincide with the points of transition between study arms could influence results. Finally, while the primary focus of the study is on demand-side barriers to timely vaccinations, participating health facilities may encounter vaccine stock-outs and power outages, resulting in vaccination activities being deferred until vaccine stocks are received or power is restored. We plan to monitor these events along with mHealth system and mobile network metrics (e.g., message transmission errors, calls dropped) during the study period. By characterizing, and if possible, addressing the limitations of our study, we will provide critical information for the feasibility of scaling up reminders and/or incentives to the regional or national levels in low-resource settings such as Tanzania.

With the continuing diffusion of mobile phones and the ongoing development of mHealth technologies, adaptive programming in the form of dynamic reminder systems and variable incentive structures has become a feasible reality even in low-resource settings. Our study sheds light on the demand-side barriers to vaccinations and the synergistic potential of combining mHealth reminders and incentives for improving vaccination coverage and timeliness. The results have implications for the design and potential efficacy of other subsidy or incentive programs aimed at improving health and health-seeking behaviors.

### Trial status

The protocol was approved by the Institutional Review Boards at Duke University, the University of South Carolina, and the National Institute for Medical Research in Tanzania and registered in ClinicalTrials.gov (Protocol NCT03252288, September 28, 2017) on August 17, 2017 (retrospectively registered).

Recruitment was ongoing at the time of manuscript submission. At the time of manuscript revision, end-line surveys were completed for 325 of 412 participants enrolled in the study.

## Additional files

Additional file 1: **Table S1**. Content of SMS reminders. (PDF 65 kb)

Additional file 2: **Table S2**. WHO Trial Registration Data Set. (PDF 66 kb)

Additional file 3: **Table S3**. SPIRIT 2013 checklist: recommended items to address in a clinical trial protocol and related documents. (PDF 92 kb)

## Data Availability

Not applicable.

## Abbreviations

BCG: Bacillus Calmette-Guerin
CFT: Conditional financial transfer
DPT: Diphtheria-pertussis-tetanus
EDD: Expected delivery date
IRB: Institutional Review Board
IVD: Immunizations and Vaccine Development (program)
km: Kilometer
LMIC: Low- and middle-income country
MCV: Measles-containing vaccine
mHealth: Mobile health
MR: Measles-rubella
OPV(*x*): Oral polio vaccine; *x* indicates the first, second, or third dose in vaccine series. Zero indicates dose due at birth
PCV(*x*): Pneumococcal vaccine; *x* indicates the first, second, or third dose in the vaccine series
Penta(*x*): Pentavalent vaccine (DPT-*Haemophilus influenzae* B-Hepatitis B); *x* indicates the first, second, or third dose in the vaccine series
Rota(*x*): Vaccine for rotavirus infection; *x* indicates the first or second dose in the vaccine series
SMS: Short message service (text messages)
TSH: Tanzania Shilling
USSD: Unstructured Supplementary Service Data
