# Supplementary material for: Do mobile phone-based reminders and conditional financial transfers improve the timeliness of childhood vaccinations in Tanzania? Study protocol for a quasi-randomized controlled trial
Source: Trials. 2019 Jul 4;20:397. doi: 10.1186/s13063-019-3430-4 (PMC6611039; doi:10.1186/s13063-019-3430-4)
Supplement: Supplementary file 1 — Table S1. Content of SMS reminders. (PDF 65 kb) [file 13063_2019_3430_MOESM1_ESM.pdf]

Table S1. Content of SMS reminders

	Recipient phone: Mother or Father		Recipient phone: Anyone else	
**Description**	**English**	**Kiswahili**	**English**	**Kiswahili**
Reminder, 1 week and 1 day prior to a scheduled vaccination	Your child is due for vaccination on [DUE_DATE]. If s/he already got vaccinated dial [USSD or CARRIER_SPECIFIC PHONE].	Mtoto wako anatakiwa kupata chanjo tarehe [DUE_DATE]. Akishapata chanjo piga [USSD or CARRIER_SPECIFIC PHONE].	Please remind [MOTHER_NAME] that her child is due for vaccination on [DUE_DATE]. If s/he already got vaccinated dial [USSD or CARRIER_SPECIFIC PHONE].	Tafadhali mkumbushe [MOTHER_NAME] kuwa mtoto wake anatakiwa kuchanjwa tarehe [DUE_DATE]. Akishapata chanjo piga [USSD or CARRIER_SPECIFIC PHONE].
Follow-up reminder, 14 days after a scheduled vaccination (if no vaccination report has been received)	Did your child receive the vaccination that was scheduled for [DUE_DATE]? If s/he did not get vaccinated, take him/her before [DUE_DATE+28 DAYS]. If s/he got vaccinated dial [USSD or CARRIER_SPECIFIC PHONE].	Je mtoto wako amepata chanjo aliyopangiwa tarehe [DUE_DATE]? Kama hajachanjwa, mpeleke kabla ya tarehe [DUE_DATE+28 DAYS]. Akichanjwa piga [USSD or CARRIER_SPECIFIC PHONE].	Did [MOTHER_ NAME] take her child to get vaccinated on [DUE_DATE]? If not, she should take him/her before [DUE_DATE+28 DAYS]. If s/he got vaccinated dial [USSD or CARRIER_SPECIFIC PHONE].	Je [MOTHER_NAME] amempeleka mtoto kuchanjwa tarehe [DUE_DATE]? Kama sio, ampeleke kabla ya tarehe [DUE_DATE+28 DAYS]. Akichanjwa piga [USSD or CARRIER_SPECIFIC PHONE].
Reminder with full incentive, 1 week and 1 day prior to a scheduled vaccination	Your child is due for vaccination on [DUE_DATE]. If s/he receives the vaccination on time you will receive SH2000. If s/he already got vaccinated dial [USSD or CARRIER_SPECIFIC PHONE].	Mtoto wako anatakiwa kupata chanjo tarehe [DUE_DATE]. Akipata chanjo kwa wakati utapokea Sh2000. Akishapata chanjo piga [USSD or CARRIER_SPECIFIC PHONE].	Please remind [MOTHER_NAME] that her child is due for vaccination on [DUE_DATE]. She will receive Sh2000 if s/he is vaccinated on time. If s/he already got vaccinated dial [USSD or CARRIER_SPECIFIC PHONE].	Tafadhali mkumbushe [MOTHER_NAME] kuwa mtoto wake anatakiwa kuchanjwa tarehe [DUE_DATE]. Atapokea Sh2000 akichanjwa kwa wakati. Akishapata chanjo piga [USSD or CARRIER_SPECIFIC PHONE]..
Follow-up reminder with half the incentive, 14 days after a scheduled vaccination (if no vaccination report has been received)	Did your child receive the vaccination that was scheduled for [DUE_DATE]? If s/he did not get vaccinated, take him/her before [DUE_DATE+28 DAYS] and you will receive SH1000. If s/he got vaccinated dial [USSD or CARRIER_SPECIFIC PHONE].	Je mtoto wako amepata chanjo aliyopangiwa tarehe [DUE_DATE]? Kama hajachanjwa, mpeleke kabla ya tarehe [DUE_DATE+28 DAYS] utapokea Sh1000. Akichanjwa piga [USSD or CARRIER_SPECIFIC PHONE].	Did [MOTHER_ NAME] take her child to get vaccinated on [DUE_DATE]? If not, she should take him/her before [DUE_DATE+28 DAYS] to receive Sh1000. If s/he got vaccinated dial [USSD or CARRIER_SPECIFIC PHONE].	Je [MOTHER_NAME] amempeleka mtoto kuchanjwa tarehe [DUE_DATE]? Kama sio, ampeleke kabla ya tarehe [DUE_DATE+28 DAYS] atapokea Sh1000. Akichanjwa piga [USSD or CARRIER_SPECIFIC PHONE].
