# Supplementary material for: Do mobile phone-based reminders and conditional financial transfers improve the timeliness of childhood vaccinations in Tanzania? Study protocol for a quasi-randomized controlled trial
Source: Trials. 2019 Jul 4;20:397. doi: 10.1186/s13063-019-3430-4 (PMC6611039; doi:10.1186/s13063-019-3430-4)
Supplement: Supplementary file 2 — Table S2. WHO Trial Registration Data Set. (PDF 66 kb) [file 13063_2019_3430_MOESM2_ESM.pdf]

Table S2: WHO Trial Registration Data Set

Data category	Information
Primary registry and trial identifying number	ClinicalTrials.gov<br>NCT03252288
Date of registration in primary registry	August 17, 2017
Secondary identifying numbers	N/A
Source(s) of monetary or material support	John E. Fogarty International Center (FIC); Duke Global Health Institute
Primary sponsor	Duke University, USA
Secondary sponsor(s)	University of South Carolina, USA, National Institute for Medical Research, Tanzania
Contact for public queries	Lavanya Vasudevan, PhD<br>Ph: 9196131423<br>lavanya.vasudevan@duke.edu<br>Jan Ostermann, PhD<br>Ph: 8037778747<br>jano@mailbox.sc.edu
Contact for scientific queries	Lavanya Vasudevan, PhD<br>Ph: 9196131423<br>lavanya.vasudevan@duke.edu<br>Jan Ostermann, PhD<br>Ph: 8037778747<br>jano@mailbox.sc.edu
Public title	mHealth-Assisted Conditional Cash Transfers to Improve Timeliness of Vaccinations (MINT)
Scientific title	mHealth-Assisted Conditional Cash Transfers to Improve Timeliness of Vaccinations (MINT)
Countries of recruitment	Tanzania
Health conditions or problems studied	Childhood vaccinations
Intervention(s)	Behavioral: Reminders<br>Behavioral: Conditional financial transfers
Key inclusion and exclusion criteria	Ages Eligible for Study: 16 Years and older<br>Sexes Eligible for Study: Female<br>Accepts Healthy Volunteers: No<br>Inclusion criteria: Pregnant, last trimester, access to mobile phone<br>Exclusion criteria: Cognitive impairment, unwillingness to receive study communication and reminders via mobile phone
Study type	Interventional (Clinical Trial)
Date of first enrolment	August 15, 2017
Target sample size	600
Recruitment status	Completed
Primary outcome(s)	1. Timely vaccination visits<br>[Time Frame: Up to 6 months after birth]<br>Number of vaccination visits within 4 weeks of scheduled visit dates
Key secondary outcomes	1. Number of vaccinations received<br>[Time Frame: Up to 6 months after birth]<br>Number of vaccinations received<br>2. Number of timely vaccinations received<br>[Time Frame: Up to 6 months after birth]<br>Number of vaccinations received within 4 weeks of scheduled vaccination dates
